# Formulation-Process Screening of a High-Oil-Fraction Ovotransferrin–Lysozyme Concentrated Dispersion Stabilized by Flaxseed Gum/Xanthan Gum Blends and High-Pressure Homogenization

**DOI:** 10.3390/foods15132386

**Published:** 2026-07-04

**Authors:** Jingyi Zhang, Anjia Huang, Yinlong Lian, Juan Chen, Xue Zhao, Dongrong Zhu, Chenggang Cai

**Affiliations:** 1 School of Biological and Chemical Engineering, Zhejiang University of Science and Technology, Hangzhou 310023, China; 222403857027@zust.edu.cn (J.Z.); 212403817032@zust.edu.cn (A.H.); 222503860032@zust.edu.cn (Y.L.); 222503860009@zust.edu.cn (J.C.); 222503855005@zust.edu.cn (X.Z.); 2 Zhejiang AGS Biotechnology Co., Ltd., Huzhou 313100, China; cxlldz@163.com

**Keywords:** ovotransferrin, lysozyme, high-oil-fraction concentrated dispersion, flaxseed gum, xanthan gum, OVT immunoreactivity, formulation screening

## Abstract

Ovotransferrin (OVT) and lysozyme (LYS) are food-derived proteins with reported bioactive properties, but results from high-oil-fraction dispersions require cautious interpretation when phase type and undiluted particle size are not independently verified. This work screened a low-temperature OVT-LYS concentrated dispersion in which freeze-drying was omitted from the final preparation stage. LYS was first incorporated into a β-cyclodextrin/trehalose-protected LYS complex (LPC). A sequential one-factor-at-a-time design was used to screen hydrocolloid type, homogenization parameters, and flaxseed gum/xanthan gum mass ratio. Qualitative optical microscopy, zeta potential, creaming stability, pH/temperature tolerance, OVT immunoreactivity, and DLS data obtained after 1000-fold dilution were used as descriptors. Among the six tested hydrocolloids, flaxseed gum (FG) showed the most favorable overall single-hydrocolloid performance. High-pressure homogenization at 1000 bar for 1.5 min, corresponding to approximately three estimated equivalent passes, was selected as a practical processing condition within the tested range. A flaxseed gum:xanthan gum mass ratio of 1:2 showed the most favorable balance between pH/temperature tolerance and OVT immunoreactivity retention among the tested binary blends. Because LYS bioactivity assays, phase-type verification, DLS dilution-gradient validation, and undiluted particle-size measurement were not performed, the results support bounded formulation-screening evidence, rather than verified phase structure, retained LYS activity, or universal optimal parameters.

## 1. Introduction

Colloidal delivery systems and related concentrated dispersions are widely used to deliver bioactive ingredients, modulate texture, and enhance food functionality. For protein-based systems, the main challenge is to combine interfacial stability, process adaptability, and retention of measurable protein indicators in the liquid state [1]. Protein–polysaccharide complexes are useful for this purpose because proteins can adsorb at oil–water interfaces and reduce interfacial tension, while polysaccharides can limit collision, gravitational separation, and local aggregation through steric and bulk-phase effects [1,2]. This combined stabilization is especially relevant for high-oil-fraction systems that cannot be interpreted as conventional dilute model dispersions.

Egg-white proteins are widely used in the food industry because of their broad availability, high nutritional value, and desirable foaming, gelling, and emulsifying properties [3]. Among them, ovotransferrin (OVT) is an iron-binding glycoprotein in egg white that exhibits promising nutritional, antioxidant, antimicrobial, and interfacial properties [3,4]. Nevertheless, OVT is sensitive to processing and storage conditions. Its structural features and measurable immunoreactivity can be perturbed by thermal stress, interfacial rearrangement, and prolonged liquid storage, which limits its direct application in non-freeze-dried liquid systems [4]. Previous studies have shown that OVT-derived structures or OVT-polysaccharide interfacial assemblies can markedly improve the interfacial stability and carrier performance of structured protein-based dispersion systems, indicating that interface engineering based on OVT is both feasible and meaningful [5,6].

Lysozyme (LYS) is another important egg-white protein with reported enzymatic and antimicrobial properties. In this work, LYS was not evaluated by an enzymatic or antimicrobial assay. LYS-related claims are therefore limited to structural incorporation as a protected complex. Like many labile proteins, LYS is susceptible to deactivation during high-shear processing, interfacial exposure, and long-term storage. Sugars, cyclodextrins, and polyols can alleviate conformational disturbances during freeze-drying and storage, and disaccharides are particularly effective in maintaining structural integrity [7,8,9]. Preparing a β-cyclodextrin/trehalose-protected LYS complex before dispersion preparation was therefore used as a structural protection strategy, not as evidence of retained LYS activity.

Hydrocolloid type and compatibility with the protein phase strongly affect the final dispersion. Polysaccharides differ in molecular flexibility, charge density, solution conformation, and thickening ability. These differences influence interfacial layer formation, near-interface interactions, and coalescence or flocculation during storage [1,10]. Homogenization is another critical processing factor. High-pressure homogenization can accelerate oil-phase disruption and interfacial reconstruction, whereas excessive intensity may promote re-aggregation or recoalescence by generating too much new interface and increasing short-time collisions. The present study therefore treats homogenization as a bounded process-screening variable, not as a route to a universal optimum or to the smallest DLS-derived apparent size.

Among the candidate hydrocolloids, flaxseed gum (FG) and xanthan gum (XG) deserve particular attention. FG is a natural anionic heteropolysaccharide extracted from flaxseed mucilage. It contains neutral and acidic polysaccharide fractions that can provide hydration, interfacial assistance, and bulk-phase thickening. XG is an anionic extracellular polysaccharide produced by Xanthomonas species and has a rigid chain conformation, high water-binding capacity, and strong pseudoplastic thickening behavior. FG can assist interfacial regulation, while XG can reinforce matrix structuring and resistance to separation [11,12]. FG/XG combinations have been reported to generate denser microstructures in protein systems and improve rheological stability [11]. Because protein–polysaccharide complexes are also governed by pH, component ratio, and preparation route, systematic screening of hydrocolloid type, blending ratio, and processing conditions remains necessary [13].

Although considerable progress has been made in protein–polysaccharide concentrated dispersions, most previous work has focused on model colloidal systems, powder reconstitution systems, or formulation screening centered on a single physical stability index [1,5,6]. In contrast, systematic studies on low-temperature OVT-LYS concentrated dispersions, whose final fabrication step avoids freeze-drying, and which simultaneously consider physical stability, environmental tolerance, and OVT immunoreactivity retention, remain limited. For such systems, reproducible screening requires coordinated consideration of interfacial construction, continuous-phase support, processing intensity, and measurable protein indicators.

Accordingly, the present study focused on a high-oil-fraction OVT-LYS concentrated dispersion containing OVT, LPC, chitosan, low-molecular-weight food emulsifiers, camellia oil, and hydrocolloids. Four operational terms were used throughout the manuscript: LPC, aqueous premix, coarse dispersion, and final high-oil-fraction concentrated dispersion. The experimental logic followed a process–structure–property framework. Formulation composition and homogenization setting were treated as process variables. Qualitative microscopy, zeta potential, and creaming behavior were used as structural or stability descriptors. OVT immunoreactivity retention was evaluated as the protein-specific performance property. DLS after 1000-fold dilution was retained only as a same-protocol auxiliary descriptor. The aim was to identify the best-performing formulation-process combination within the tested OFAT design, rather than to establish a global optimum or a verified mechanism.

## 2. Materials and Methods

### 2.1. Materials and Reagents

Ovotransferrin (OVT) was extracted and purified in-house from fresh egg white (obtained from eggs purchased from a local supermarket) and used as the main interfacial protein. Lysozyme (LYS; purity ≥ 90%) was purchased from Shanghai Macklin Biochemical Co., Ltd. (Shanghai, China). Food-grade hydrocolloid stabilizers, including xanthan gum (XG), flaxseed gum (FG), gellan gum (GeG), guar gum (GuG), locust bean gum (LBG), and konjac glucomannan (KGM), were obtained from Henan Gaocui Biotechnology Co., Ltd. (Zhengzhou, China). Food-grade emulsifiers, glycerol monostearate and Tween 80, were purchased from Shanghai Macklin Biochemical Co., Ltd. (Shanghai, China). Food-grade camellia oil was supplied by Jiangxi Qinglong High-Tech Oils & Fats Co., Ltd. (Yichun, China).

An enzyme-linked immunosorbent assay (ELISA) kit for chicken ovotransferrin (catalog no. CB10103-Ch) was purchased from Shanghai Jianglai Biotechnology Co., Ltd. (Shanghai, China). All other reagents were of analytical grade. Ultrapure water was produced by a Millipore system (18.2 MΩ·cm at 25 °C).

### 2.2. Preparation and Process Screening of OVT-LYS Concentrated Dispersions

**Figure 1:**
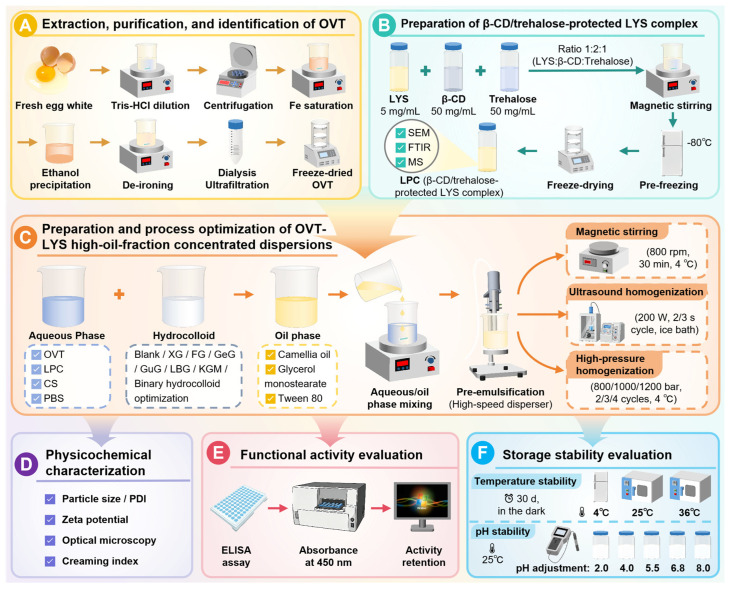
Schematic overview of the preparation, screening, and evaluation of OVT-LYS concentrated dispersions. Abbreviations: OVT, ovotransferrin; LYS, lysozyme; LPC, β-cyclodextrin/trehalose-protected LYS complex; β-CD, β-cyclodextrin; CS, chitosan; PBS, phosphate-buffered saline; FG, flaxseed gum; XG, xanthan gum. The schematic is used to define the preparation sequence only, and does not independently verify dispersion-phase type.

This section describes the workflow used to prepare, screen, and evaluate OVT-LYS concentrated dispersions, as schematically illustrated in Figure 1.

#### 2.2.1. Extraction, Purification, and Identification of OVT

Extraction and purification of ovotransferrin were optimized from reported procedures for egg-white-derived OVT to minimize processing-induced structural alteration before ELISA-based OVT assessment [14]. Fresh egg white was diluted 1:1 (*v*/*v*) with pre-cooled 0.05 M Tris-HCl buffer (pH 8.0) at 4 °C and mixed thoroughly, followed by centrifugation at 6000 rpm for 20 min using a benchtop high-speed refrigerated centrifuge (H2-16KR, Hunan Kecheng Instrument Equipment Co., Ltd., Changsha, China) to remove ovomucin. The supernatant then underwent iron saturation, gradient ethanol precipitation to remove impurities and to sediment the target protein, re-dissolution of the precipitate, de-ironing with an anion-exchange resin, dialysis desalting, ultrafiltration concentration, and vacuum freeze-drying using a freeze dryer (SCIENTZ-10N/C, Ningbo Scientz Biotechnology Co., Ltd., Ningbo, China) to obtain OVT. Protein concentration was determined using the bicinchoninic acid (BCA) assay, while purity and identity were assessed by sodium dodecyl sulfate–polyacrylamide gel electrophoresis (SDS-PAGE) and matrix-assisted laser desorption/ionization time-of-flight tandem mass spectrometry (MALDI-TOF/TOF MS, autoflex TOF/TOF, Bruker Daltonics, Bremen, Germany) fingerprinting. Data acquisition was performed using flexControl v3.4.169.5, and protein identification was conducted using flexAnalysis software 3.4. The purified OVT was stored at −20 °C in sealed, light-protected conditions.

#### 2.2.2. Preparation and Structural Characterization of the β-Cyclodextrin/Trehalose-Protected LYS Complex (LPC)

The β-cyclodextrin/trehalose-protected LYS complex is hereafter abbreviated as LPC. LPC denotes only the pre-encapsulated LYS-containing powder prepared before dispersion preparation; it is not an independent dispersion phase. To prepare LPC, LYS was combined with β-cyclodextrin (β-CD) and trehalose using a stepwise stirring–ultrasound-assisted method. The procedure was based on reported cyclodextrin inclusion methods and the protective effect of trehalose during protein freeze-drying [7,15]. All solutions were freshly prepared in 0.01 mol/L phosphate-buffered saline (PBS, pH 7.4), including 5 mg/mL LYS solution, 50 mg/mL β-CD solution, and 50 mg/mL trehalose solution.

Equal volumes of the LYS and β-CD solutions were mixed and magnetically stirred at 4 °C and 500 rpm for 15 min using a constant temperature magnetic stirrer (B11-2, Shanghai Sile Instrument Co., Ltd., Shanghai, China) to allow preliminary encapsulation of LYS by β-CD. An additional equal volume of β-CD solution and an equal volume of trehalose solution were then added so that the final volume ratio of LYS:β-CD:trehalose was 1:2:1. The mixture was further stirred for 1.5 h at the same temperature and speed, with ultrasound dispersion at 200 W for 5 min every 30 min using an ultrasonic cell disruptor (SCIENTZ-950E, Ningbo Scientz Biotechnology Co., Ltd., Ningbo, China) to promote uniform formation and structural stabilization of the encapsulation system [7,16]. The resulting encapsulation solution was pre-frozen at −80 °C for 2 h and then vacuum freeze-dried for 48 h under a vacuum of ≤10 Pa with a cold-trap temperature of −80 °C. The obtained freeze-dried powder of the inner complex was stored at −20 °C in sealed, light-protected conditions [7,15].

Scanning electron microscopy (SEM, SU1510, Hitachi High-Technologies, Tokyo, Japan) was used to compare the microstructures of free LYS and the encapsulated complex [7]. After sputter-coating with a 10 nm gold layer, images were acquired at an accelerating voltage of 15 kV under different magnifications to analyze structural changes before and after encapsulation.

Fourier-transform infrared spectroscopy (FTIR, Vertex 70, Bruker Optics, Ettlingen, Germany) was used to characterize molecular structural changes and host–guest inclusion interactions [7]. Samples and controls were prepared by the KBr pellet method. Spectra were recorded from 4000 to 400 cm^−1^ at a resolution of 4 cm^−1^ with 32 scans, using pure KBr as the background. Changes in characteristic absorption peaks were compared, to support formation of the encapsulation complex.

The same MALDI-TOF/TOF MS instrument described in Section 2.2.1 was used to compare mass spectral profiles and primary-structure-related signals of LYS before and after encapsulation [17].

#### 2.2.3. Preparation of OVT-LYS Concentrated Dispersions

The preparation and sampling points were defined using four operational terms. LPC denotes the protected LYS-containing powder described in Section 2.2.2. Aqueous premix denotes the mixture of LPC suspension, OVT solution, chitosan solution, and the hydrocolloid slot. Coarse dispersion denotes the oil-containing mixture after oil addition and pre-dispersion, but before final protectant incorporation. Final high-oil-fraction concentrated dispersion denotes the product after protectant addition and 15 min stirring. Unless otherwise stated, all physicochemical and ELISA samples were collected from this final dispersion. Final fabrication without freeze-drying refers only to the transition from the aqueous premix/coarse dispersion to the final high-oil-fraction concentrated dispersion; OVT and LPC preparation still involved separate freeze-drying steps. All solutions were pre-cooled and handled at 4 °C before mixing. During HPH, the cooling module was set at 4 °C. Continuous in-line sample-temperature recording was not performed, so this value is reported only as the instrument setting, and transient sample heating during homogenization cannot be excluded. No conductivity or dye-staining phase-type test was performed. The product is therefore described as a high-oil-fraction concentrated dispersion, rather than as a verified phase-type system. The oil fraction was 73.8% before protectant addition and 58.4% in the final product. The first value approaches the close-packing region often discussed for high-internal-phase emulsions, while the final product remains a concentrated, potentially gel-like dispersion [18]. Cooling was provided by the homogenizer’s built-in recirculating cooling module.

Stock solutions and oil phase were prepared as follows. The aqueous stocks included 0.5% (*w*/*v*) chitosan solution (degree of deacetylation ≥ 95%, viscosity 100–200 mPa·s, prepared in 0.5% acetic acid), 10 mg/mL OVT solution, 20 mg/mL LPC suspension, and a protectant solution. The LPC suspension was prepared by adding freeze-dried LPC powder at 125% of the theoretical loading, vortex-resuspending it in PBS, centrifuging at 5000× *g* for 5 min at 4 °C, and collecting the supernatant. The protectant solution contained 7.5% (*w*/*v*) trehalose and 1.5% (*v*/*v*) glycerol in pre-cooled 0.01 mol/L PBS (pH 7.4). The oil phase contained 45 mL camellia oil and 1.4 g of a glycerol monostearate/Tween 80 blend (1:1, *w*/*w*; mixed HLB approximately 9.4). The blend was dissolved at 60 °C and 500 rpm for 15 min and then cooled to 4 °C. Hydrocolloid stock solutions (2%, *w*/*v*) of XG, FG, GeG, GuG, LBG, and KGM were prepared as described below. The blank used PBS/water in the hydrocolloid slot. Except for GeG, which was prepared in ultrapure water, hydrocolloids were prepared in ultrapure water containing 0.145 M NaCl, heated to 80 °C for GeG, 40 °C for XG and KGM, 45 °C for FG, and 37 °C for GuG and LBG, and then cooled to 4 °C. Comparisons involving GeG should therefore be interpreted as formulation-screening results under the stated preparation protocol.

For each 77 mL final dispersion, 6 mL LPC suspension, 3 mL OVT solution, 6 mL chitosan solution, and 1 mL hydrocolloid solution (or PBS/water for the blank) were mixed at 4 °C and 500 rpm for 40 min to form 16 mL aqueous premix. The oil phase (45 mL) was then introduced portionwise under the same temperature and stirring conditions, to obtain a coarse dispersion. The coarse dispersion was pre-dispersed in an ice-water bath for 15 s at 10,000 rpm, using a cantilever high-speed disperser (THF 500-12G, Tuohe Electromechanical Technology (Shanghai) Co., Ltd., Shanghai, China).

It was then processed under the selected dispersion or homogenization condition. Finally, 16 mL protectant solution was added, and the mixture was stirred at 4 °C and 500 rpm for 15 min to obtain the final high-oil-fraction concentrated dispersion. The final composition and calculation notes are summarized in Table 1.

#### 2.2.4. Screening of Key Parameters of the Dispersion System

A sequential one-factor-at-a-time (OFAT) screening strategy was used to compare OVT-LYS concentrated dispersions within the tested formulation and processing ranges. This design can identify best-performing conditions in the present experimental space and evaluate individual factors. It cannot determine a mathematical global optimum or quantify interactions among hydrocolloid type, hydrocolloid ratio, oil fraction, and homogenization intensity. Qualitative microstructure, zeta potential, creaming index, and OVT immunoreactivity retention were selected as the principal descriptors. DLS-derived apparent size and PDI after 1000-fold dilution were recorded only as same-protocol auxiliary descriptors. Formulation superiority was therefore not assigned on the basis of DLS size alone.

Six single hydrocolloid solutions, namely XG, FG, GeG, GuG, LBG, and KGM (all 2%, *w*/*v*), were first prepared. Their flow behavior and macroscopic appearance were compared using a qualitative inversion-flow test, in which identical tubes containing the hydrocolloid solutions were inverted for the same observation period and the relative movement of the solution front was visually recorded. This test was used only as a simple macroscopic indicator of relative flow resistance and weak-gel tendency, not as quantitative rheology. Each hydrocolloid solution was then used as the sole stabilizer in the dispersion system, while all other formulation variables and the basic preparation procedure described in Section 2.2.3 were kept constant. The dispersions were prepared by magnetic stirring at 800 rpm for 30 min under continuous temperature control at 4 °C, and preliminary screening was carried out with microstructural uniformity as a qualitative criterion, without quantitative inference from the inversion-flow test or microscopy.

Candidate hydrocolloids were further evaluated by preparing dispersions under temperature-controlled conditions and subjecting them to HPH at 1000 bar for 1.5 min. The homogenizer was operated by pressure and processing time, rather than by a preset pass counter. Estimated equivalent passes were therefore calculated using Equation (1). This calculation was used only to make the processing intensity traceable; it does not indicate direct pass-count control. Microstructural morphology was used as a qualitative screening observation. Under this protocol, FG showed better visual dispersion than the other single hydrocolloids, whereas GeG showed the least favorable performance. Five parallel treatments were then applied using the same base formulation. Zeta potential and creaming behavior were used as primary process-matching descriptors, and DLS apparent size was retained as an auxiliary descriptor [10]. The treatments were: (1) magnetic stirring at 800 rpm for 30 min in a 4 °C thermostatic water bath; (2) ultrasound homogenization at 200 W with a working/pause cycle of 2 s/3 s under ice-water cooling using an ultrasonic cell disruptor (SCIENTZ-950E, Ningbo Scientz Biotechnology Co., Ltd., Ningbo, China); and (3) HPH at 800, 1000, and 1200 bar for 1.0, 1.5, and 2.0 min, respectively, under a 4 °C cooling setting using a high-pressure homogenizer (AH-LAB, Antos Nano Technology (Suzhou) Co., Ltd., Suzhou, China). For HPH, each sample was continuously recirculated back into the same feed vessel, and the feed vessel was manually mixed with a glass rod to reduce local concentration differences. In Equation (1), Q is the manufacturer’s nominal throughput (10 L/h), t is the processing time (h), and V is the total processed sample volume entering HPH (L); thus, Q × t/V is a residence-volume estimate, rather than a directly counted pass number.

(1) Estimated passes = Q × t/V.

Based on the secondary screening results, FG was used as the core hydrocolloid to establish the experimental and control groups. Dispersions were prepared under the fixed HPH condition of 1000 bar for 1.5 min, corresponding to approximately three estimated equivalent passes by Equation (1). Binary hydrocolloid formulations were compared within the tested ratios using qualitative microscopy, zeta potential, creaming index, and OVT immunoreactivity, with DLS apparent size retained only as an auxiliary observation. The FG:XG ratios of 1:1, 2:1, and 1:2 represented equal-mass, FG-rich, and XG-rich blends, respectively, under the same total hydrocolloid addition in the hydrocolloid slot. The reverse-control group was an FG:GeG blend at a mass ratio of 1:1, and a hydrocolloid-free blank group was also included. All references to best-performing or selected formulations refer only to the tested OFAT design, and do not exclude other optima outside this space.

### 2.3. Basic Physicochemical Characterization and Microstructural Observation of Dispersions

#### 2.3.1. Determination of Particle Size, Polydispersity Index, and Zeta Potential

Particle size, PDI, and zeta potential were determined, with slight modifications to reported methods [19,20,21,22,23], using a Zetasizer Nano ZS (Malvern Panalytical, Worcestershire, UK). Before analysis, aliquots of the final high-oil-fraction concentrated dispersion were mixed with pre-cooled 0.01 mol/L PBS (pH 7.4) at 4 °C to obtain 1000-fold diluted measurement dispersions. Dilution was used to reduce optical density and minimize multiple scattering, which is a recognized concern in DLS measurements of concentrated suspensions and dispersions [20,21,23]. DLS reports an intensity-weighted hydrodynamic diameter derived from Brownian diffusion. It does not directly measure microscopic dispersed-domain diameter. Because none of the following were performed: 100-, 500-, and 1000-fold dilution-gradient validation, undiluted laser-diffraction measurement, and microscopic size quantification, DLS results are reported only as apparent sizes of diluted measurement dispersions. Particle size and PDI were measured at 4 °C, whereas zeta potential was measured at 25 °C on the same diluted samples. All determinations were carried out using the instrument default settings in three independent replicates.

#### 2.3.2. Optical Microscopy

Optical microscopy (ECLIPSE Ci, Nikon, Tokyo, Japan) was used to observe dispersion microstructure and to visualize dispersion and aggregation characteristics [19]. Samples were taken from the final high-oil-fraction concentrated dispersion after protectant addition and 15 min stirring at 4 °C. Images were acquired under 10× and/or 40× objective magnification, as specified in the corresponding figure captions. Scale bars were inserted using the calibrated microscope software (version 5.42.07) and standardized as 100 μm for 10× images and 20 μm for 40× images. The micrographs were used for qualitative comparison of dispersion uniformity, coarse structures, and local aggregation, rather than as a stand-alone quantitative particle-size method or proof of phase type. Gray-level or contrast differences among panels were not used as quantitative evidence, and visually similar micrographs were not used alone to rank formulations.

#### 2.3.3. Determination of Creaming Index

The creaming index was determined by a static standing method with reference to published procedures [19]. Equal volumes of dispersion samples were transferred into graduated sealed centrifuge tubes of identical specifications, and stored under the designated conditions. At predetermined time points, the volume of the cream layer and the initial total dispersion volume were recorded. The creaming index was calculated using Equation (2).

(2) Creaming index (%) = (cream-layer volume/initial total dispersion volume) × 100.

### 2.4. Evaluation of OVT Immunoreactivity

Following the kit instructions and published ELISA quantification strategies, an enzyme-linked immunosorbent assay (ELISA) kit specific for chicken ovotransferrin was used to determine OVT immunoreactivity in the dispersions [24]. Samples were collected from the final high-oil-fraction concentrated dispersion after protectant addition and 15 min stirring at 4 °C, consistent with the physicochemical measurements. Absorbance was measured at 450 nm, using a microplate reader (Feyond-A300, Allsheng, Hangzhou, China). The ELISA-detectable OVT content and OVT immunoreactivity retention rate were calculated from the standard curve and Equation (3). The purified OVT fraction was approximately 90% pure. ELISA results were therefore interpreted as OVT-specific immunoreactivity within an OVT-rich material, rather than as evidence that minor co-purified proteins were absent from the matrix. No LYS enzymatic or antimicrobial activity assay was performed, so retained LYS enzymatic or antimicrobial function is not claimed in this work. Although the kit is specified for chicken OVT, cross-reactivity with the residual non-OVT fraction and matrix recovery in the final concentrated dispersion were not independently validated; absolute concentrations and between-formulation ratios should therefore be interpreted with this limitation.

(3) OVT immunoreactivity retention (%) = C_t_/C_0_ × 100.

### 2.5. Evaluation of Dispersion Storage Stability

#### 2.5.1. Temperature Stability

Equal volumes of dispersion samples were stored at 4, 25, and 36 °C in the dark, for 30 d. The creaming index was measured every 5 d to evaluate the effect of storage temperature on dispersion stability.

#### 2.5.2. pH Stability

Equal volumes of dispersion samples were adjusted to initial pH values of 2.0, 4.0, 5.5, 6.8, and 8.0 using 1 mol/L citric acid or 1 mol/L NaOH; they were then sealed, and stored at 25 °C in the dark for 30 d. The reported pH values refer to the initial adjusted pH before storage; pH drift during storage was not monitored. The creaming index was measured every 5 d, and phase separation was observed simultaneously, to evaluate dispersion stability under initial pH-adjusted conditions.

### 2.6. Data Processing and Statistical Analysis

All experiments were performed in triplicate, and results are expressed as mean ± standard deviation (mean ± SD). Statistical analyses and figure plotting were conducted using Microsoft Excel 2019 and GraphPad Prism 9.5. Differences among groups were assessed by one-way analysis of variance (ANOVA) followed by Tukey’s multiple comparison test, and *p* < 0.05 was considered statistically significant. Statistical interpretation was based on the reported significance groupings at *p* < 0.05. 

## 3. Results and Discussion

### 3.1. Structural Characterization of the Key Materials

#### 3.1.1. Purity Identification and Structural Characterization of OVT

**Figure 2:**
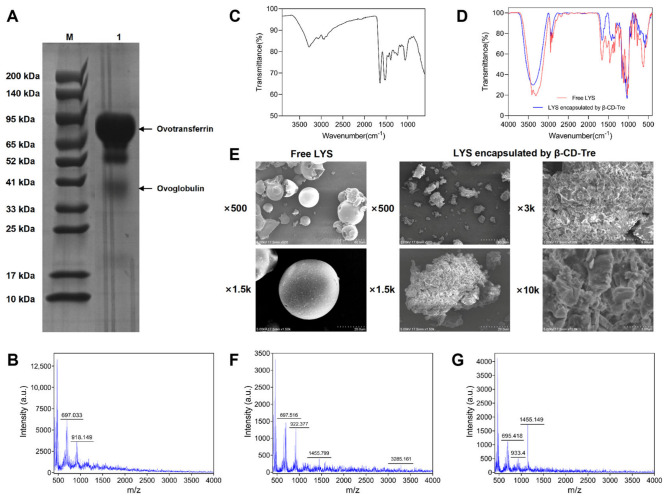
Basic structural characterization of the key functional materials. (**A**) SDS-PAGE pattern of the OVT sample (M, protein molecular-weight marker; lane 1, purified OVT sample). (**B**) MALDI-TOF/TOF MS spectrum of the OVT sample. (**C**) FTIR spectrum of the OVT sample. (**D**) Comparative FTIR spectra of free LYS and the β-cyclodextrin-trehalose-encapsulated complex. (**E**) SEM micrographs of free LYS and the β-cyclodextrin-trehalose-encapsulated complex. (**F**,**G**) MALDI-TOF/TOF MS spectra of the free LYS standard (**F**) and the β-CD-Tre-LYS complex (**G**).

The extracted and purified OVT sample was subjected to purity and structural characterization. The SDS-PAGE pattern (Figure 2A) showed a single sharp major band at 76–80 kDa, which agreed with the theoretical molecular weight of OVT and indicated a dominant OVT band with minor co-purified bands [4,14,25]. Densitometric analysis indicated that the purity of OVT reached approximately 90%. This purity was considered sufficient for formulation screening, but it was not equivalent to analytical-grade purity. A minor non-OVT fraction may therefore remain, so quantitative ratio interpretations refer to the prepared OVT-rich fraction. The ELISA kit was used to specifically quantify OVT immunoreactivity. The MALDI-TOF/TOF MS peptide mass fingerprint (Figure 2B) showed characteristic fragment peaks of OVT, including medium-mass peaks at *m*/*z* 697.033 and 918.149 and low-mass peaks at *m*/*z* 431.299, 434.220, and 449.144 [26]. These data confirmed that OVT was the major component and that the extraction process did not damage its core amino-acid sequence. FTIR analysis (Figure 2C) showed amide A and amide I/II bands consistent with reported OVT features [4,25].

#### 3.1.2. Microstructure and Encapsulation-Related Morphology of the β-CD-Tre-LYS Complex

SEM was used to characterize the microstructural differences before and after encapsulation, and thereby support LPC formation. Free LYS appeared as smooth spherical aggregates with diameters of 20–50 μm. After encapsulation with β-cyclodextrin and trehalose, the complex formed irregular porous agglomerates of 10–30 μm. At higher magnification, wrinkled and stacked lamellar structures and interconnected pores of approximately 100 nm to 5 μm were observed (Figure 2E). Cyclodextrins can bind exposed hydrophobic regions on protein surfaces through host–guest recognition and form non-covalent complexes with proteins. Such interactions often change particle morphology from regular and dense to rough, porous, and loose [16,27,28,29]. The morphological changes observed here are consistent with LPC formation and provide a structural basis for possible LYS protection during subsequent dispersion processing. They do not demonstrate retained enzymatic or antimicrobial activity.

#### 3.1.3. Molecular Structural Characterization of the β-CD-Tre-LYS Complex

FTIR was used to examine the effect of β-cyclodextrin-trehalose (β-CD-Tre) encapsulation on LYS structure (Figure 2D). Compared with free LYS, LPC showed several spectral changes associated with encapsulation. In the hydroxyl stretching region at 3200–3600 cm^−1^, the -OH stretching band shifted from 3420 to 3380 cm^−1^, and the full width at half maximum after conversion to absorbance increased from 120 to 185 cm^−1^. This change indicates formation of a new intermolecular hydrogen-bond network involving β-cyclodextrin and trehalose [28,29,30,31]. In the amide I region (1600–1700 cm^−1^), the α-helix band at 1652 cm^−1^ decreased by 23%, whereas the β-sheet band at 1630 cm^−1^ increased by 18%. This suggests secondary-structure redistribution, rather than retained LYS function [29,30,31]. In the glycosidic bond region, at 1000–1200 cm^−1^, the absorption intensity at 1085 cm^−1^ increased by 15%, which was attributed to intermolecular hydrogen bonding between the C-O-C linkage of trehalose and polar residues of LYS [29]. Overall, β-CD-Tre mainly regulated LYS structure through hydrogen-bond reconstruction and local microenvironment remodeling.

MALDI-TOF/TOF MS was further used to compare primary-structure-related signals of free LYS (Figure 2F) and LPC (Figure 2G). Characteristic LYS peaks were still detected in LPC, and their peak-position deviations were small, indicating maintenance of primary-structure-related signals after encapsulation [32,33,34]. Some higher-molecular-weight peaks became weaker or disappeared after encapsulation. This may reflect altered protein ionization efficiency and exposure state in the encapsulating matrix [32,34]. No substantial number of abnormal new impurity peaks appeared in the encapsulated sample, and interference from low-molecular-weight peaks was generally reduced [32,33]. Together with FTIR, these data suggest that β-CD-Tre and LYS formed a relatively stable encapsulation complex, mainly through non-covalent interactions such as hydrogen bonding and hydrophobic association. Retained LYS enzymatic or antimicrobial function still requires direct testing.

### 3.2. Systematic Screening of Single Hydrocolloids

**Figure 3:**
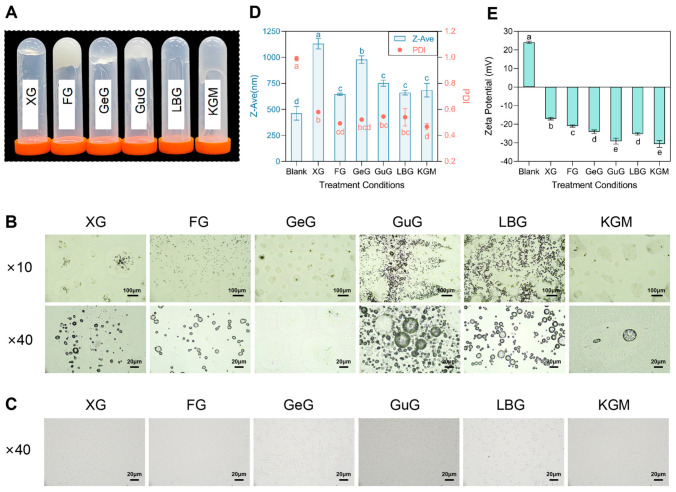
Systematic screening of single hydrocolloids. (**A**) Macroscopic appearance and qualitative inversion-flow behavior of 2% (*w*/*v*) single-hydrocolloid solutions. (**B**) Optical micrographs of dispersions stabilized by different single hydrocolloids and prepared by magnetic stirring (10× and 40×). (**C**) Optical micrographs of dispersions stabilized by different single hydrocolloids and prepared by high-pressure homogenization at 1000 bar (40×). Scale bars: 100 μm for 10× images and 20 μm for 40× images. (**D**) DLS-derived apparent Z-average hydrodynamic diameter (Z-ave) and polydispersity index (PDI) of 1000-fold diluted measurement dispersions. (**E**) Zeta potential of diluted measurement dispersions prepared from the same samples. Notes: Blank, hydrocolloid-free dispersion; XG, xanthan gum; FG, flaxseed gum; GeG, gellan gum; GuG, guar gum; LBG, locust bean gum; KGM, konjac glucomannan. Data are presented as mean ± SD (*n* = 3). Different lowercase letters indicate significant differences among groups (*p* < 0.05). In panel (**D**), blue bars represent Z-ave and red dots represent PDI. DLS data were obtained only from 1000-fold diluted measurement dispersions, and were used as auxiliary same-protocol descriptors, not as evidence of true product size.

To select a candidate hydrocolloid stabilizer, the macroscopic appearance, qualitative inversion-flow behavior, microscopy, zeta potential, and storage-related trends of six hydrocolloids were compared. DLS measurements after 1000-fold dilution were retained only as auxiliary same-protocol observations. As shown in Figure 3A, the 2% (*w*/*v*) hydrocolloid solutions differed markedly in appearance and inversion-flow behavior. These differences suggest different viscosity build-up, molecular-chain entanglement, and weak-gel tendencies. In egg-white-protein systems, added polysaccharides can affect both bulk rheology and protein rearrangement near interfaces. Macroscopic viscosity alone is therefore insufficient to predict dispersion performance [1,3].

Optical micrographs of the primary dispersions prepared by magnetic stirring (Figure 3B) showed qualitative differences in dispersion uniformity. The FG system displayed comparatively uniform visual dispersion, whereas GeG, GuG, and LBG systems exhibited more obvious coarse structures or local aggregates. After HPH at 1000 bar (Figure 3C), all systems changed visually, to varying degrees. The FG group still showed comparatively uniform morphology, while the GeG group remained the least favorable. Because microscopy was used qualitatively, these observations were evaluated together with creaming index and electrokinetic data. They were not used as stand-alone proof of dispersed-domain size or phase type. The stability of protein–polysaccharide concentrated dispersions depends on rapid rebuilding of interfacial protection and bulk structural support after disruption, not only on thickening of the matrix [2,3,5].

Figure 3D shows DLS-derived apparent size as an auxiliary descriptor. All six single hydrocolloids changed the Z-average hydrodynamic diameter (Z-ave) and PDI relative to the blank, after dilution. These values do not represent direct dispersed-domain diameters in the original product. Figure 3E shows that hydrocolloids shifted the zeta potential from positive in the blank dispersion to negative in all hydrocolloid-containing systems. This result suggests participation of anionic hydrocolloids in the interface or near-interface region. A more negative potential did not directly translate into better overall performance. System behavior was therefore interpreted from zeta potential, creaming behavior, OVT immunoreactivity, and qualitative microscopy, with DLS used secondarily [3,13].

Overall, the macroscopic flow behavior, qualitative microscopy, zeta potential, and storage-related observations indicate that FG showed the best potential as a single hydrocolloid stabilizer within the tested protocol. FG was therefore selected as the core hydrocolloid for subsequent binary formulation design. This selection was not based on DLS apparent size as a decisive index. Previous studies have likewise shown that flaxseed gum can improve protein-dispersion stability by increasing viscosity, suppressing creaming, and assisting interfacial stabilization [12,35].

### 3.3. Systematic Screening of Homogenization Parameters

**Figure 4:**
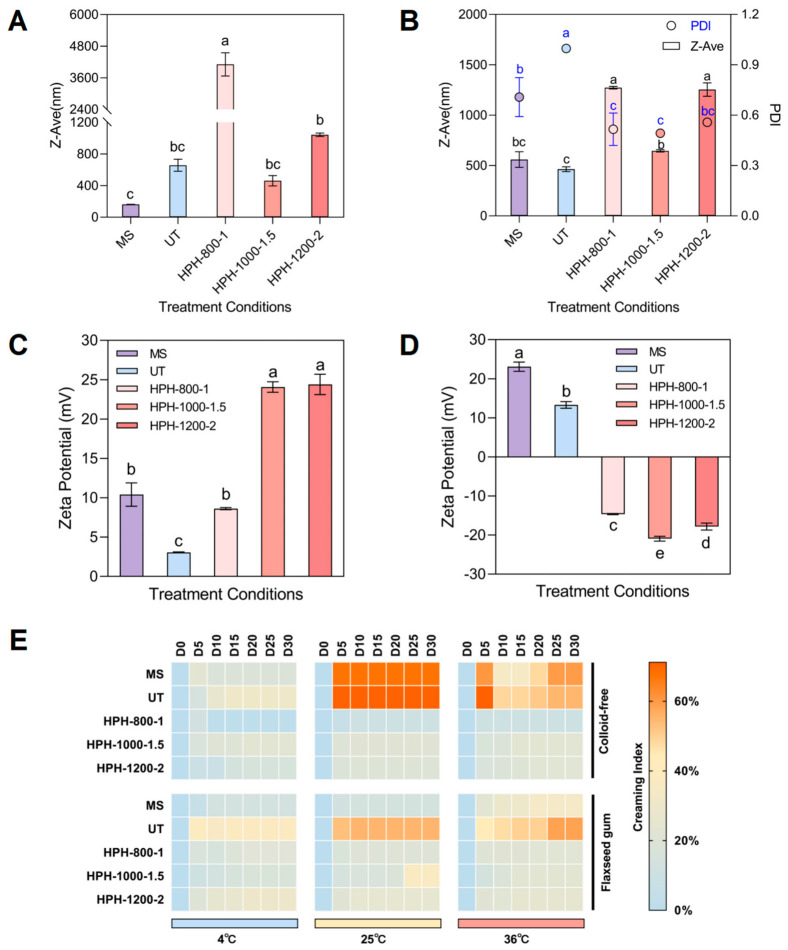
Systematic screening of homogenization parameters. (**A**) Effect of different homogenization treatments on the DLS-derived apparent Z-average hydrodynamic diameter (Z-ave) of blank dispersions after 1000-fold dilution. (**B**) Effect of different homogenization treatments on the DLS-derived apparent Z-average hydrodynamic diameter (Z-ave) and polydispersity index (PDI) of FG-stabilized dispersions after 1000-fold dilution. (**C**) Effect of different homogenization treatments on the zeta potential of blank dispersions. (**D**) Effect of different homogenization treatments on the zeta potential of FG-stabilized dispersions. (**E**) Heat map of the creaming index of blank dispersions and FG-stabilized dispersions prepared by different homogenization processes during storage at different temperatures. Notes: MS, magnetic stirring; UT, ultrasound homogenization; HPH-800-1, HPH-1000-1.5, and HPH-1200-2 denote high-pressure homogenization at 800, 1000, and 1200 bar for 1.0, 1.5, and 2.0 min, respectively. Estimated equivalent passes were calculated using Equation (1), corresponding to approximately 2, 3, and 4 estimated passes. This was an approximation, rather than direct pass-count control. Detailed parameters are given in Section 2.2.4. Data are presented as mean ± SD (*n* = 3). Different lowercase letters indicate significant differences among groups (*p* < 0.05).

After FG had been identified as the candidate hydrocolloid, the effects of different homogenization procedures were examined in the blank and FG-stabilized dispersions. For the blank group, Figure 4A shows that homogenization method strongly affected the DLS-derived apparent Z-ave after dilution. These values are interpreted only as diluted-state auxiliary descriptors. Figure 4C shows that increasing treatment intensity altered the zeta potential of the blank dispersion, indicating changes in interfacial or near-interface charge state. Stronger HPH therefore changed electrokinetic properties, but did not necessarily improve storage behavior. Oil-domain disruption, aggregation, and recoalescence may have competed during processing. Overly mild treatment may not support effective interfacial reconstruction. Excessive treatment, especially 1200 bar for 2.0 min, may generate an abundant new interface faster than stabilizers can cover it, and may increase collision frequency under short-time high-shear conditions [2,10,13].

For FG-stabilized dispersions, process differences were more discriminative. As shown in Figure 4B, ultrasound treatment (UT) produced a low apparent Z-ave, but the highest PDI under the diluted measurement condition. This indicates broad structural heterogeneity after dilution, not superior product size. The apparent Z-ave values of HPH-800-1 and HPH-1200-2 were both higher than that of HPH-1000-1.5. Figure 4D further shows that HPH-1000-1.5 had a large absolute zeta-potential value, only slightly lower than that of HPH-1200-2. Together with storage behavior, these results support a moderate HPH window. In this window, interfacial reconstruction and matrix stabilization were better coordinated than under lower or higher pressure. Because 1000 bar for 1.5 min represented approximately three estimated equivalent passes by Equation (1), the process selection should be interpreted as a practical time-pressure condition within the tested OFAT design, not as a preset machine cycle number or a global optimum. Similar behavior has been reported for protein–polysaccharide systems, where excessive homogenization can increase collision frequency and destabilization, despite improving some electrokinetic descriptors [10,13].

The storage heat map (Figure 4E) further demonstrates the fact that process selection could not rely on apparent initial size data. For blank dispersions, MS, UT, and some HPH treatments showed more pronounced increases in creaming index at 25 and 36 °C. For FG-stabilized dispersions, HPH-1000-1.5 maintained lower creaming indices across storage temperatures and showed better long-term stability. Accordingly, process selection was based on the combined electrokinetic, qualitative-microscopy, and storage-stability evidence. HPH-1000-1.5 was selected as the best-performing homogenization condition within the tested range for subsequent binary hydrocolloid design. The limited advantage of HPH-1200-2 supports the interpretation that excessive pressure may promote secondary aggregation or recoalescence, rather than continuous refinement [10,13].

### 3.4. Screening of Binary Hydrocolloid Formulation and Evaluation of OVT Immunoreactivity

**Figure 5:**
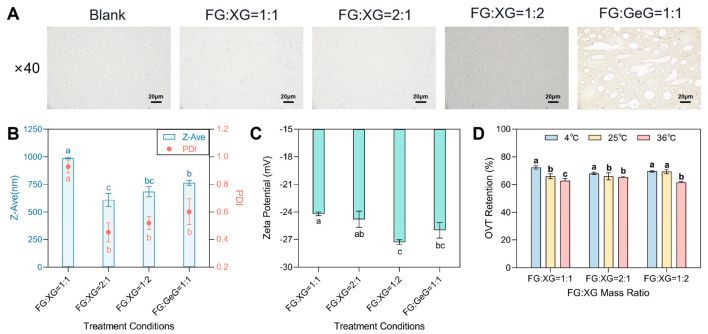
Screening of binary hydrocolloid formulations and evaluation of OVT immunoreactivity. (**A**) Optical micrographs (40×) of dispersions stabilized by different binary hydrocolloid formulations under the selected homogenization condition. Scale bars: 20 μm for 40× images. (**B**) DLS-derived apparent Z-average hydrodynamic diameter (Z-ave) and polydispersity index (PDI) of 1000-fold diluted measurement dispersions; these data were not used as primary evidence for formulation superiority. (**C**) Zeta potential of the diluted measurement dispersions. (**D**) Retention of OVT immunoreactivity in dispersions stabilized by different binary hydrocolloid formulations after storage for 30 d at 4, 25, and 36 °C. Notes: FG:XG = 1:1, 2:1, and 1:2 denote flaxseed gum-to-xanthan gum mass ratios of 1:1, 2:1, and 1:2, respectively; FG:GeG = 1:1 denotes a flaxseed gum-to-gellan gum mass ratio of 1:1. Data are presented as mean ± SD (*n* = 3). Different lowercase letters indicate significant differences among groups under the same test condition (*p* < 0.05). DLS apparent size in panel (**B**) was auxiliary, and was not used as the primary formulation-selection criterion.

On the basis of the selected homogenization condition, different FG:XG and FG:GeG binary systems were compared. The 1:1, 2:1, and 1:2 FG:XG ratios represented equal, FG-dominant, and XG-dominant blending patterns at the same total hydrocolloid level. This design compared FG-related interfacial or dispersion support with XG-related matrix structuring. As shown in Figure 5A, all three FG:XG ratios exhibited relatively good qualitative dispersion, whereas FG:GeG = 1:1 showed obvious coarse, irregular, and elongated structures. Figure 5B reports DLS-derived apparent size only as a non-decisive auxiliary descriptor. FG:XG = 2:1 yielded the lowest apparent Z-ave and PDI after dilution, but this result was not used alone to determine superiority. Figure 5C shows that FG:XG = 1:2 had the largest absolute negative zeta potential, indicating a stronger electrostatic barrier at the interface or near-interface region. Blend screening therefore reflected a trade-off among interfacial contribution, matrix structuring, and storage behavior. FG and XG can act synergistically in protein systems by densifying microstructural networks and improving rheological support, although the best ratio remains system-dependent [11,12]. In concentrated high-oil-fraction dispersions, such matrix and interfacial effects may be more important than small differences in diluted-state apparent size [18].

The OVT immunoreactivity retention shown in Figure 5D did not strictly follow the DLS-derived apparent size trend. All three FG:XG formulations showed decreased OVT immunoreactivity retention after storage for 30 d at 4, 25, and 36 °C, but the extent of decrease differed among formulations. FG:XG = 1:1 showed slightly higher OVT immunoreactivity retention at 4 °C. FG:XG = 1:2 showed the highest retention at 25 °C. At 36 °C, both FG:XG = 2:1 and FG:XG = 1:2 were higher than the 1:1 formulation. Because ELISA reflects recognizable OVT epitopes, these results indicate that binary hydrocolloids may help preserve OVT conformation and epitope accessibility beyond their effect on diluted-state apparent size. In protein–polysaccharide concentrated dispersions, a poorly organized interfacial layer or local aggregation may accelerate conformational exposure during storage. A suitably organized composite interface and surrounding matrix may buffer environmental stress and preserve OVT immunoreactivity more effectively [5,6,13]. The exact Tukey-adjusted *p* values for the major between-formulation and across-temperature comparisons are listed in Table 2.

Exact Tukey-adjusted *p* values for the major OVT-retention comparisons in Figure 5D are listed in Table 2.

**Table 2:** Exact Tukey-adjusted *p* values for OVT-immunoreactivity comparisons in Figure 5D.

Between-Formulation Comparison	4 °C	25 °C	36 °C
FG:XG = 1:1 vs. 2:1	<0.0001	0.9989	0.0015
FG:XG = 1:1 vs. 1:2	0.0002	<0.0001	0.1915
FG:XG = 2:1 vs. 1:2	0.0718	<0.0001	<0.0001
Temperature comparison	FG:XG = 1:1	FG:XG = 2:1	FG:XG = 1:2
4 °C vs. 25 °C	<0.0001	0.0063	0.9617
4 °C vs. 36 °C	<0.0001	0.0002	<0.0001
25 °C vs. 36 °C	<0.0001	0.5416	<0.0001

Overall, Figure 5B–D show that FG:XG = 2:1 was advantageous mainly for auxiliary DLS apparent size after dilution, whereas FG:XG = 1:2 provided the most favorable balance when zeta potential, creaming stability, and OVT immunoreactivity retention were prioritized. At 25 °C, FG:XG = 1:2 gave the highest OVT immunoreactivity retention and the largest absolute negative zeta potential among the binary systems. The higher XG proportion may therefore have strengthened electrostatic and matrix support in this high-oil-fraction dispersion. Together with the temperature- and pH-tolerance results, FG:XG = 1:2 was selected as the best-performing binary hydrocolloid formulation within the tested OFAT ratios. This choice reflects the strongest combined performance among the measured descriptors under the present OFAT design, although individual treatments led in some indices. Compared with reported OVT-based Pickering or oleogel-in-water systems, which mainly emphasize interfacial stabilization and delivery performance [19,36,37], the present system also considered OVT immunoreactivity after storage, and avoided assigning unverified phase identity. This context-dependent interpretation is also consistent with a recent review emphasizing OVT digestive stability, cross-matrix interactions, and targeted food applications [38].

### 3.5. Comprehensive Evaluation of Storage Stability and Environmental Tolerance

**Figure 6:**
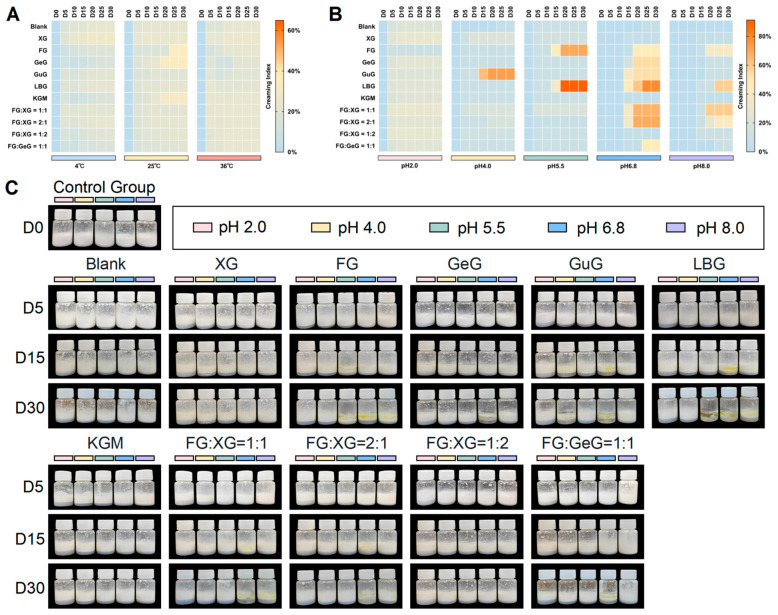
Comprehensive evaluation of storage stability and environmental tolerance of the dispersions. (**A**) Heat map of the creaming index of blank dispersions, single-hydrocolloid dispersions, and binary-hydrocolloid dispersions during storage at 4, 25, and 36 °C under the selected homogenization condition. (**B**) Heat map of the creaming index of blank dispersions, single-hydrocolloid dispersions, and binary-hydrocolloid dispersions during storage under different initial pH-adjusted conditions. (**C**) Macroscopic appearance changes of the dispersions during storage under different initial pH-adjusted conditions. Notes: Blank denotes the hydrocolloid-free dispersion. The heat-map color changes from blue to orange as the creaming index increases. The reported pH values refer to the initial adjusted pH before storage. The creaming index was calculated as cream-layer volume divided by the initial total dispersion volume × 100%. These tests evaluate storage behavior of the high-oil-fraction concentrated dispersion, and do not verify phase type.

After identification of the selected process and binary formulation within the tested ranges, the robustness of the final dispersion was evaluated under different temperatures and initial pH values. Figure 6A shows that the creaming indices of all systems remained relatively low at 4 °C, indicating that low temperature retarded phase separation. As the temperature increased to 25 and 36 °C, differences among systems became more apparent. The blank dispersion and some single-hydrocolloid dispersions were more prone to creaming at medium and high temperatures. Binary hydrocolloid systems were generally more stable than single-hydrocolloid systems. Among them, FG:XG = 1:2 maintained comparatively low creaming indices at both 25 and 36 °C, and showed more favorable long-term storage stability within the tested groups. The advantage of the binary strategy lay mainly in sustained interfacial support and matrix structuring during storage, rather than in DLS-derived apparent initial-size control. Concentrated high-oil-fraction systems often show rheological and separation behavior that differs from dilute model dispersions [18].

Figure 6B,C further reveal an initial-pH-sensitive stability window. The most pronounced destabilization occurred mainly in the pH 4.0–6.8 range, and the differences among hydrocolloids were magnified in this interval. For example, the GuG system showed obvious creaming at pH 4.0, whereas the FG, and, especially, the LBG systems, became more unstable at pH 5.5–6.8. By contrast, the binary systems, especially FG:XG = 1:2, remained comparatively stable over initial pH 2.0–8.0 and maintained low creaming levels under neutral and mildly alkaline conditions. The macroscopic appearance shown in Figure 6C was consistent with the heat-map trends. Some single-hydrocolloid samples displayed evident phase separation or bottom sedimentation during storage, whereas the binary systems retained better visual integrity. Because independent phase-type verification and rheological characterization were not performed, these observations are interpreted as storage behavior of a high-oil-fraction concentrated dispersion, not as a complete mechanistic description of a verified phase-type system. Protein–polysaccharide complexes can undergo charge redistribution, chain-conformation adjustment, and altered interfacial adsorption at different initial pH values, so instability can concentrate within specific pH windows, rather than only under extreme acid or alkaline conditions [6,13].

Taken together, Figure 5 and Figure 6 show that the screening pathway has methodological value within a bounded formulation space. The study first identified a candidate stabilizing framework by screening single hydrocolloids. It then avoided excessive processing by selecting a moderate HPH window. Finally, binary hydrocolloid design was used to balance zeta potential, storage stability, environmental tolerance, and OVT immunoreactivity retention. These findings should be interpreted within the specific context of the OVT-LYS high-oil-fraction concentrated dispersion and its OFAT formulation-processing design. The OFAT design can reveal individual factor effects and identify best-performing conditions within the tested range, but it cannot resolve higher-order interactions. The DLS method provides only apparent hydrodynamic diameters under identical 1000-fold dilution. It cannot verify the true size distribution of the original product without dilution-gradient or undiluted measurement. The phase type was not independently verified, and the final oil fraction of 58.4% supports interpretation as a concentrated, potentially gel-like dispersion, rather than a conventional dilute model system. Future studies should include phase-type analysis, dilution-series or undiluted size measurements, rheology, protein conformational spectroscopy, digestion or oxidation tests, and direct LYS enzymatic or antimicrobial activity assays.

## 4. Conclusions

This study established a bounded formulation-screening pathway for a high-oil-fraction OVT-LYS concentrated dispersion. The workflow covered single-hydrocolloid screening, homogenization selection, binary formulation design, and environmental-tolerance evaluation. Among six single hydrocolloids, FG showed the most favorable overall qualitative dispersion, electrokinetic behavior, and storage-related trends under the same protocol. FG was therefore used as the core stabilizing framework. DLS-derived apparent size after 1000-fold dilution was retained only as an auxiliary same-protocol descriptor, and was not used to determine product size or formulation superiority.

At the process level, HPH-1000-1.5 (1000 bar, 1.5 min; approximately three estimated equivalent passes) provided the best balance between electrokinetic behavior and storage stability within the tested conditions. Higher intensity, represented by HPH-1200-2, did not provide a stronger recommendation, and may promote secondary aggregation or recoalescence. Further binary screening showed that FG:XG = 1:2 exhibited the most favorable combined temperature/pH tolerance and OVT immunoreactivity retention. It was therefore selected as the best-performing formulation within the tested OFAT design. This selection does not represent a global optimum, and cannot resolve interactions among formulation variables.

The conclusions remain bounded by the available evidence. Final fabrication without freeze-drying refers only to the final dispersion preparation step, not to every upstream material-preparation step. LYS was structurally incorporated as LPC, but its residual enzymatic or antimicrobial activity was not measured. Phase type was not independently verified by conductivity or dye tests. DLS size data were obtained only from 1000-fold diluted measurement dispersions. Therefore, the study supports a conservative formulation-process screening route, rather than a verified phase-type system, a universal optimum, retained LYS enzymatic or antimicrobial function, or a fully validated mechanism. Further work should combine phase-type verification, dilution-series or undiluted particle-size characterization, rheology, conformational characterization, LYS activity assays, and digestion behavior, to clarify stabilization mechanisms and application potential.

## Figures and Tables

**Table 1:** Preparation stages and final composition of the OVT-LYS high-oil-fraction concentrated dispersion under the constant phase-ratio framework.

Stage/Slot	Composition or Amount	Definition/Function
Operational terms	LPC; aqueous premix; coarse dispersion; final dispersion	Terms used to trace preparation and sampling.
LPC suspension	20 mg/mL β-CD/trehalose-protected LYS complex in PBS; 6 mL	Protected LYS-containing component.
OVT solution	10 mg/mL OVT solution; 3 mL	Protein quantified by ELISA.
CS and hydrocolloid slot	6 mL of 0.5% (*w*/*v*) CS + 1 mL of 2% (*w*/*v*) hydrocolloid or PBS/water	Fixed aqueous slot for formulation comparison.
Oil phase	45 mL camellia oil + 1.4 g GMS/Tween 80 (1:1, *w*/*w*)	High-oil-fraction component.
Protectant solution	16 mL PBS with 7.5% (*w*/*v*) trehalose and 1.5% (*v*/*v*) glycerol	Aqueous post-homogenization addition.
Final sampling point	Aqueous premix:oil phase:protectant = 16:45:16; final volume = 77 mL	Samples collected after protectant addition and 15 min stirring.

Notes: The final oil fraction was calculated as 45/(16 + 45 + 16) = 58.4% (*v*/*v*), ignoring small solute and density effects; before protectant addition, the oil fraction was 45/(16 + 45) = 73.8% (*v*/*v*). The final emulsifier level was approximately 1.82% (*w*/*v*). The hydrocolloid slot contained 20 mg total hydrocolloid, and binary FG:XG ratios refer to this fixed mass. The protectant solution was not treated as a separate phase, and no phase-inversion or phase-type assay was performed. DLS/zeta potential, microscopy, creaming index, and ELISA samples were collected from the final dispersion; DLS was conducted after 1000-fold dilution as an auxiliary descriptor.

## Data Availability

The datasets generated and analyzed during the current study are available from the corresponding author upon reasonable request.
